# Get strong to fight childhood cancer - an exercise intervention for children and adolescents undergoing anti-cancer treatment (FORTEe): Rationale and design of a randomized controlled exercise trial

**DOI:** 10.1186/s12885-025-14489-y

**Published:** 2025-08-07

**Authors:** Marie Astrid Neu, Elias Dreismickenbecker, Francesca Lanfranconi, Sandra Stössel, Adriana Balduzzi, Peter Wright, Stan Windsor, Joachim Wiskemann, Inaam El-Rajab, Alejandro Lucia, Carmen Fiuza-Luces, Rodolf Mongondry, Martin Kaj Fridh, Filippo Spreafico, Barbara Konda, Lidija Kitanovski, Barbara Heißerer, Marco Polak, Tobias Baader, Wilhelm Bloch, Miriam Götte, on behalf of Network ActiveOncoKids, Katie Rizvi, on behalf of Youth Cancer Europe, Christian Ruckes, Norbert W. Paul, Joerg Faber, on behalf of the FORTEe Consortium, Ameli Schwalber, Olivia Pérol, Hanne Bækgaard Larsen, Tommaso Pietro Moriggi, William Zardo, Amandine Bertrand, Lena Wypyrsczyk, Mareike Kühn, Abigale Robinson, Heidi Diel

**Affiliations:** 1 Childhood Cancer Center Mainz, University Medical Center of the Johannes Gutenberg-University Mainz, Mainz, 55131 Germany; 2 Centro Maria Letizia Verga, Fondazione Monza E Brianza Per Il Bambino E La Sua Mamma, Monza, 20900 Italy; 3 Pediatric Department, Fondazione IRCCS San Gerardo dei Tintori and School of Medicine and Surgery, Milano-Bicocca University, Monza, 20900 Italy; 4 Department of Sport and Health Science and Social Work, Oxford Brookes University, Oxford, OX3 0BP UK; 5 Department of Medical Oncology, Working Group Exercise Oncology, Heidelberg University Hospital and National Center for Tumor Diseases, a partnership between DKFZ and University Medical Center Heidelberg, Heidelberg, 69120 Germany; 6 Department of Sports Sciences, Faculty of Medicine, Health and Sports, Universidad Europea de Madrid, Madrid, 28670 Spain; 7 Hospital 12 de Octubre Research Institute (“imas12”)‚ Physical Activity and Health Laboratory, Madrid, 28041 Spain; 8 Prevention Cancer Environment Department, Centre de Lutte Contre le Cancer Léon Bérard, Lyon, 69373 France; 9 Department of Pediatrics and Adolescent Medicine, University Hospital Copenhagen, Rigshospitalet, Copenhagen, 2100 Denmark; 10 Pediatric Oncology Unit, Fondazione IRCCS Istituto Nazionale Dei Tumori, Milan, 20133 Italy; 11 Forma 3D Ltd., Ljubljana, 1000 Slovenia; 12 Division of Pediatrics, Department of Haematooncology, University Medical Center Ljubljana, Ljubljana, 1000 Slovenia; 13 concentris research management gmbh, Fürstenfeldbruck, 82256 Germany; 14 Nurogames GmbH, 50676 Cologne, Germany; 15 Pixformance Sports GmbH, Dallgow-Döberitz, 14624 Germany; 16 Department of Molecular and Cellular Sport Medicine at the Institute of Cardiology and Sports Medicine, German Sport University Cologne, Cologne, 50933 Germany; 17 University Hospital Essen, West German Cancer Center, Essen, 45122 Germany; 18 Youth Cancer Europe, Cluj-Napoca, 400 372 Romania; 19 University Medical Center of the Johannes Gutenberg-University Mainz, Interdisciplinary Centre for clinical Studies (IZKS), Mainz, 55131 Germany; 20 University Medical Center of the Johannes Gutenberg-University Mainz, Institute for the History, Philosophy and Ethics of Medicine, Mainz, 55131 Germany

**Keywords:** Childhood cancer, Pediatric Oncology, Exercise intervention, Physical activity, Randomized controlled trial, Cancer-related fatigue, Supportive Care, Training

## Abstract

**Background:**

Despite substantial advances in treatment, children and adolescents with cancer continue to face high morbidity and health issues, including cancer-related fatigue, treatment-related complications, and physical inactivity. Integrating exercise into pediatric oncology care has emerged as a promising approach to mitigate these burdens during cancer treatment. While preliminary data support its potential to reduce treatment-related side effects and enhance quality of life, robust evidence -especially from large, multicenter trials- remains limited.

**Methods:**

The FORTEe trial is a randomized, controlled, multicenter trial evaluating a personalized and standardized exercise intervention powered to include 450 children, adolescents, and young adults undergoing cancer treatment across ten centers in Europe. The trial aims to provide high-quality evidence for integrating precision exercise therapy as part of standard care. Participants are randomly assigned to either the exercise intervention group, receiving a tailored, supervised 8–10 weeks lasting exercise program, or the control group, receiving usual care. The exercise program includes endurance, strength, flexibility, and balance training, adapted to each patient’s age, fitness, and cancer treatment phase. Exercise sessions are intended to take place 3–5 times a week with moderate intensity, with both frequency and intensity adapted to the clinical condition of the individual. Digital tools and telehealth solutions support the intervention, allowing for both in-person and remote training.

**Discussion:**

With a target enrolment of 450 patients, the FORTEe trial will be one of the largest interventional studies in pediatric exercise oncology. Given that childhood cancer is a rare disease, this sample size is only achievable through a multicenter approach. Enhancing statistical power, the large sample will enable more robust analyses of the intervention’s effects in a diverse population across multiple European centers.

**Conclusion:**

As a progress beyond the current state-of-the-art, FORTEe has the ambition to implement pediatric exercise oncology as an evidence-based treatment option for all childhood cancer patients, ultimately integrating it as a standard into clinical practice worldwide.

**Trial registration:**

The FORTEe trial was prospectively registered in the German Clinical Trials Register (DRKS00027978) on 28 January 2022 and on ClinicalTrials.gov (NCT05289739) on 21 March 2022.

**Supplementary Information:**

The online version contains supplementary material available at 10.1186/s12885-025-14489-y.

## Introduction

Despite substantial advances in treatment, children and adolescents with cancer continue to experience high morbidity and health issues, notably due to treatment‐related side effects such as physical inactivity and cancer‐related fatigue (CRF). Although childhood cancer is a rare disease, it remains the leading cause of death from non‐communicable diseases in European children [1, 2]. Over the past decades, survival rates have substantially improved [3], with more than 80% of childhood cancer patients now surviving beyond five years [4–6]. However, patients and survivors face short- and long-term risks including cardiovascular and metabolic complications, which are often exacerbated by physical inactivity and reduced fitness levels. These factors contribute to diminished health-related quality of life (HRQoL) and increased long-term healthcare needs [7, 8]. During cancer treatment, children with cancer frequently become physically inactive due to disease burden, hospitalization, therapy-induced toxicities, and psychosocial sequelae [9, 10]. In this context, there is growing recognition of the need to optimize supportive care in pediatric oncology. In recent years, the field of exercise oncology has emerged as a promising area, investigating structured exercise as an adjunct to conventional cancer care. In adult cancer, exercise interventions have shown benefits in reducing CRF, enhancing physical fitness and function, improving psychological function as well as improving HRQoL [11]. CRF, a multidimensional syndrome characterized by persistent exhaustion unrelieved by rest, profoundly affects the physical, mental, and social well-being of cancer patients [12]. Despite its substantial impact, the underlying mechanisms of CRF are still not fully understood [13], and treatment approaches continue to evolve [14]. Notably, CRF is increasingly recognized as a prevalent and debilitating side effect in children and adolescents undergoing cancer therapy [15–17].

However, exercise guidelines and evidence generated in adult populations [18] are not directly applicable to children due to physiological, developmental, and psychosocial differences [19]. Childhood cancer encompasses a heterogeneous spectrum of malignancies and treatment regimens, each requiring individualized approaches to physical activity interventions.

Moreover, clinical research in pediatric oncology faces unique challenges compared to adult settings. The rarity of childhood cancer considerably limits achievable sample sizes in clinical trials, making robust, large-scale studies difficult [20]. Additionally, the treatment of children requires special considerations, as their developing organ systems are more susceptible to treatment-related toxicities [21]. Pediatric trials must also account for family-centered care models, as children depend on caregivers for support during treatment, which can influence adherence and intervention feasibility [22].

Furthermore, ethical and regulatory requirements for research involving minors are particularly stringent due to their vulnerability and ongoing physiological and psychosocial development. These factors necessitate careful adaptation of study designs, risk–benefit assessments, and consent procedures to ensure that trials are both ethical and effective [23]. As a result, pediatric trials must be well-structured and age-appropriate, specifically tailored to the unique needs of pediatric oncology patients while ensuring both safety and efficacy when evaluating exercise interventions.

Emerging evidence supports the feasibility, safety, and potential benefits of exercise interventions in children with cancer [24–35], as demonstrated by monocentric randomized controlled trials (RCTs) such as the MUCKI [36] and PAPEC trials [26]. Nonetheless, high-quality, large-scale data remain scarce, and standardized exercise programs are not yet routinely integrated into pediatric oncology care. Addressing this gap requires robust, multicenter randomized controlled trials that evaluate both efficacy and implementation strategies [37–39].

The FORTEe trial is a multinational RCT designed to assess the effects of a precision-based exercise intervention on CRF in children and adolescents undergoing cancer treatment. The primary objective is to determine whether structured exercise training can reduce CRF, measured by the Pediatric Quality of Life Inventory™ (PedsQL™) Multidimensional Fatigue Scale [40]. Secondary objectives include evaluating the impact on HRQoL, mental health, resilience, physical fitness, body composition and biomarkers. We hypothesize that the FORTEe intervention will reduce CRF and improve a range of physical and psychosocial outcomes, supporting the integration of personalized exercise therapy into standard pediatric oncology care.

## Materials and methods

### Overview of study design

The FORTEe trial is a multi-center RCT within the Horizon 2020 funded FORTEe research project “Get strong to fight childhood cancer– An exercise intervention for children and adolescents undergoing anti-cancer treatment”. Ten recruitment centers are based in seven European countries: Germany, Italy, Spain, France, United Kingdom, Denmark and Slovenia (see Table 1). The study design builds on insights from the monocentric RCT MUCKI [36].

**Table 1:** Overview of participating institutions, including location details and assigned acronyms

**Participant name**	**City**	**Country**	Acronym
University Medical Center of the Johannes Gutenberg-University Mainz Childhood Cancer Center Mainz	Mainz	Germany	UMC-Mainz
Heidelberg University Hospital and NCT Heidelberg (a partnership between DKFZ and University Medical Center Heidelberg) Hopp Children´s Cancer Center (KiTZ) Department of Pediatric Oncology, Hematology and Immunology, Heidelberg University Hospital	Heidelberg	Germany	UKHD
Centre de Lutte Contre le Cancer Leon Berard Pediatric Hematology and Oncology Institute (IHOPe) jointly with the Lyon Hospices Civils	Lyon	France	CLB
Oxford Brookes University in collaboration with Oxford University Hospitals NHS Foundation Trust John Radcliffe Hospital, Oxford Churchill Hospital, Oxford	Oxford	United Kingdom	OBU
Fondazione Monza e Brianza per Il Bambino e La Sua Mamma Pediatric Department, Fondazione IRCCS San Gerardo dei Tintori Centro Maria Letizia Verga Haemato-Oncology Unit and the Transplant Centre	Monza	Italy	MBBM
Region Hovedstaden University Hospital Copenhagen Rigshospitalet Department of Pediatrics and Adolescent Medicine	Copenhagen	Denmark	RegionH
Universidad Europea de Madrid in cooperation with: Hospital Infantil Universitario Niño Jesús, Madrid Hospital Universitario 12 de Octubre, Madrid	Madrid	Spain	UEM
Fondazione IRCCS Istituto Nazionale dei Tumori Pediatric Oncology Unit	Milan	Italy	INT
University Medical Center Ljubljana Department of Haematology and Oncology in Cooperation with Forma 3D Ltd., Ljubljana	Ljubljana	Slovenia	UKCL Forma 3D
University Hospital Essen West German Cancer Center Clinic for Pediatrics III, Hematology and Oncology	Essen	Germany	UKESSEN
German Sport University Cologne ^a^	Cologne	Germany	DSHS Koeln

^a^no recruiting site, scientific partner in the trial (biomarker project)

**Fig. 1:**
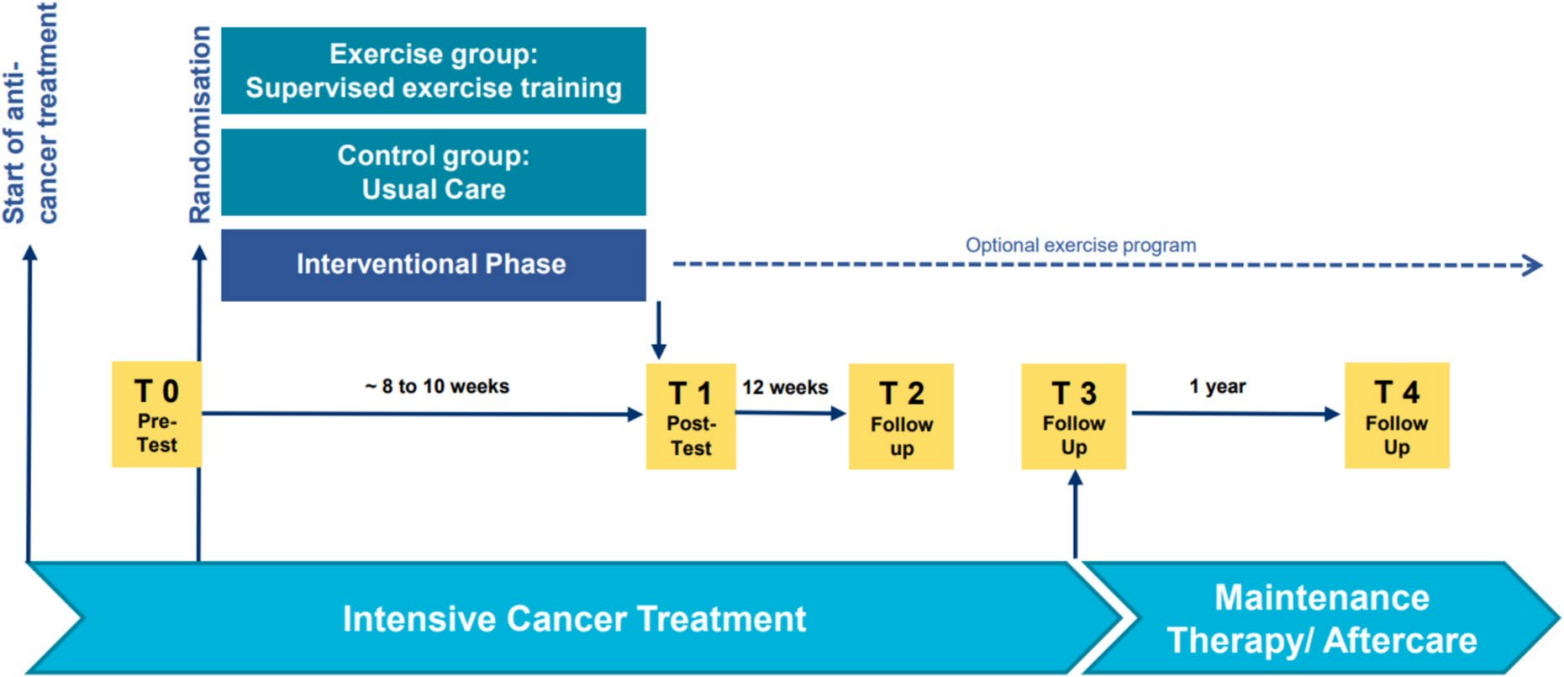
Study design of the randomized controlled FORTEe trial. The FORTEe trial evaluates the effects of a supervised exercise intervention in childhood cancer patients undergoing intensive treatment. After baseline assessments at T0 (pre-test), participants are randomized to either the exercise group (receiving supervised exercise training) or the control group (receiving usual care). The interventional phase lasts approximately 8–10 weeks and concludes with T1 (post-test). This is followed by three follow-up assessments: T2 (12 weeks after T1), T3 (at the end of intensive cancer treatment), and T4 (12 months after T3). If there are less than four weeks inbetween T2 and T3, only T2 will take place. In patients receiving allogeneic stem cell transplantation, T3 is scheduled six months post-transplant (day 0). After the interventional phase, participants may voluntarily continue with an optional, unsupervised exercise program during maintenance therapy or aftercare.

Participants of the FORTEe trial are randomly assigned to either the experimental group (exercise group), receiving a precision exercise therapy intervention, or the control group, receiving usual care. The exercise intervention lasts for 8 to 10 weeks, depending on the cancer treatment course. Key assessment points include T0 (pre-test), T1 (post-test) and three follow-ups (T2-T4, see Fig. 1). The pre- and post-test are scheduled in a standardized way, i.e., usually before the start of a new treatment cycle or at the time of best possible hematologic recovery and in as"fit"a condition as possible. After the intervention, all participants may join an optional exercise program at their recruitment center. Blinding is not feasible due to the nature of the intervention.

The FORTEe trial was prospectively registered in the German Clinical Trials Register (DRKS00027978) on 28 January 2022 and on ClinicalTrials.gov (NCT05289739) on 21 March 2022.

The trial protocol was developed in accordance with the Standard Protocol Items: Recommendations for Interventional Trials (SPIRIT) 2013 guidelines [41]. The completed SPIRIT checklist is provided in an additional file (see Additional file 1). The schedule of enrollment, interventions, and assessments is outlined in the SPIRIT Figure (see Additional file 2).

### Ethical and safety considerations

The FORTEe study protocol and associated documents were approved by the Ethics Committee of the Rhineland-Palatinate Chamber of Physicians, the consortium’s lead ethics committee, on 4th August 2021 (application number 2021–15904), followed by approvals from local ethics committees and regulatory authorities at each recruitment center in accordance with national and/or local requirements. The relevant ethics committees and regulatory authorities as well as trial registries will be notified of any changes or amendments to the protocol. All trial staff adhere to Good Clinical Practice, the Declaration of Helsinki, and the European General Data Protection Regulation (GDPR). A comprehensive Data Protection Concept has been developed, particularly concerning the use of digital tools during the trial. For minors, written informed consent from a legal guardian is required. Participants aged 16 and older must provide their own written consent, while children aged six and above are asked to provide documented assent where possible. Separate consent was obtained for ancillary studies, including biomarker and site-specific subprojects involving additional data or blood collection. Patients will be enrolled in the study only after providing written informed consent, following a minimum reflection period of 24 h. Consent may be withdrawn at any time without consequences.

To minimize the risk of Serious Exercise-Related health Complications (SERCs), all sessions are prepared and supervised by qualified exercise professionals, considering each participant’s daily clinical condition. Medical clearance is obtained before each session to ensure participant safety. The detailed recommendations for medical clearance and criteria for adapting exercise are provided in an additional file (see Additional file 3). SERCs are reported via specific electronic case report form including information such as date/onset, grade, case description, relation to exercising, potential SERC treatment/actions taken and outcome. An additional file shows the SERC code groups, SERC codes and SERC grades used in FORTEe (see Additional file 4). An evaluation on causality and expectedness will be performed for each documented SERC.

### Eligibility, recruitment and stratified randomization

Eligible participants are children, adolescents and young adults aged 4 to 21 years diagnosed with cancer and undergoing chemotherapy and/or radiotherapy. At each recruiting center, all newly diagnosed patients are screened for eligibility by study staff and informed about the FORTEe trial as early as possible following diagnosis. Recruitment was planned to occur during the initial phase of cancer treatment—when patients typically receive more intensive, predominantly intravenous, chemotherapy and/or radiotherapy. Prior to the start of patient enrolment, all staff involved at the recruiting sites received standardized training provided by the coordinating team. Participant recruitment was conducted between April 2022 and October 2024. In addition to the primary eligibility criteria, patients must meet further inclusion criteria: suitability as determined by the treating team, availability of written informed consent (and documented assent for children when appropriate), sufficient proficiency in the national language or English, and not being in the terminal phase of the disease. Exclusion criteria are provided in an additional file (see Additional file 5).

Following enrolment and completion of the baseline pre-test (T0), a centralized, computerized, stratified randomization allocates participants in a 1:1 ratio to the exercise or control group. Stratification is based on four criteria: i) the clinical recruiting center, ii) the category of childhood cancer diagnosis (classified according to the International Classification of Childhood Cancer, third edition [42], as blood cancer, malignant bone tumor, central nervous sytem (CNS) and intraspinal tumors, or Others), iii) the (intended) treatment intensity [43], and iv) the participant’s physical performance at the pre-test (measured using the Lansky/Karnofsky score). The online MARVIN database facilitates both the initiation of the randomization process and the display of the allocation results to authorized personnel at the recruitment site.

MARVIN, a validated Electronic Data Capture platform developed by XClinical, is utilized for data collection and management in this study. The database is customized for the specific needs of the trial and hosted by the Central Data Management (Zentrales Datenmanagement, ZDM) of the Society for Paediatric Oncology and Haematology (GPOH), based at Hannover Medical School (Medizinische Hochschule Hannover).

A CONSORT (Consolidated Standards of Reporting Trials) flowchart is used to summarize the eligibility and enrolment process [44].

### Intervention

Participants randomized to the exercise group will receive a supervised precision exercise training for 8 to 10 weeks during intensive cancer treatment (see Fig. 1). The intervention is continued until the post-test is completed (e.g., 12 weeks). For participants in the intervention group, exercise should be offered as frequently as possible throughout the intervention phase, ideally on a daily basis. In accordance with current literature-based recommendations, the intervention aims to deliver 3 to 5 exercise sessions per week, each lasting between 45 to 60 min. The specific duration of sessions may vary within this range, taking into account individual tolerance and clinical circumstances. The content, intensity, and duration of each session will be tailored to the patient’s functional capacity, age, fitness level, and overall health status. This individualized approach ensures that the exercise program remains safe, feasible, and beneficial across a wide range of physical abilities and treatment stages.

The program primarily consists of age-appropriate, moderate-intensity endurance, strength, flexibility, coordination/balance and gait training. Additionally, playful games are integrated into the sessions to increase motivation and enhance adherence, particularly among younger children.

Training is conducted in diverse settings, including inpatient and outpatient clinics as well as patients’ homes during outpatient stays. In inpatient settings, exercise sessions are directly supervised by qualified exercise professionals. During outpatient periods—and on occasions such as weekends during inpatient stays—, participants (if applicable, supported by their parents) exercise independently according to the recommendations of the exercise professional, and/or, depending on each recruiting center’s infrastructure with support via digital tools (e.g. augmented reality (AR) app). In addition, remote outpatient exercise training is offered using telehealth solutions supervised by FORTEe exercise professionals.

For each exercise session, both the total minutes of exercising and the time spent per exercise category (endurance, upper body strength, lower body strength, flexibility, coordination/balance, and gait) are documented. Exercise intensity is classified as moderate-to-vigorous if at least two of the following three criteria are met; otherwise, it is classified as light: i) therapist-rated intensity based on breathing rate, sweating, other signs of exertion; ii) rating of perceived exertion on the Borg 6–20 scale ≥ 12 [45]; iii) for endurance exercises: heart rate ≥ 65% of the maximum heart rate, determined either via cardiopulmonary exercise testing (CPET) or estimated using the formula HFmax = 208 − 0.7 × age [46]; for strength exercises: 1–3 repetitions performed with correct technique at a predetermined maximum repetition load [47].

In cases where a training session is terminated or cancelled, the event and the underlying reasons are documented.

The control group in this trial will not receive supervised exercise sessions during the intervention period but will continue to receive usual care, including supportive therapies as defined by site-specific standards. An additional file shows an overview about the supportive care services across the FORTEe trial sites (see Additional file 6). This design allows for a pragmatic comparison between standard care and the additional benefit of a structured, supervised exercise intervention.

The decision to use usual care as a comparator reflects both ethical and practical considerations. Given the growing but still limited integration of exercise therapy into pediatric oncology practice, usual care remains the current standard in many settings. This comparison enables evaluation of the additive effects of supervised exercise on outcomes such as physical functioning, psychosocial well-being, and treatment tolerance.

To account for unsupervised exercising, both groups are provided with an exercise diary to record frequency, type, and intensity of any voluntary exercise, as well as general physical activity in daily life. This ensures transparency regarding background activity levels and enables more accurate interpretation of the intervention’s impact.

### Outcome measures & participant timeline

The primary outcome of the FORTEe trial is CRF, measured using the PedsQL™ 3.0 Multidimensional Fatigue Scale [40, 48]. This validated questionnaire is available in age-specific versions and is completed by the participant and/or by proxy through parents or guardians. Secondary outcomes are detailed in Table 2.

**Table 2:** Overview of primary and secondary outcomes, associated measurement tools and data collection sites

Outcome		Measured by	Data collection sites
**Primary outcomes**			
*Psychosocial outcomes*			
Cancer-related fatigue		Pediatric Quality of Life Inventory™ (PedsQL™) 3.0 Multidimensional Fatigue Scale [40]	All Sites
**Secondary outcomes**			
*Psychosocial outcomes*			
Health-related quality of life		PedsQL™ 4.0 Generic Core Scales questionnaire [49],	All sites
		PedsQL™ 3.0 Cancer Module [40]	All sites
Resilience		Mainzer Resilience Scale for childhood cancer (MRScc)^©^	All sites
		Brief Resilience Scale (BRS) [50],	All sites
		Child & Youth Resilience Measure-Revised (CYRM-R) [51]	All sites
Self-efficacy		General self-efficacy scale (GSE) [52]	All sites
		Physical exercise self-efficacy scale (PESE) [53]	All sites
Mental health		WHO (Five) Well-Being Index [54]	All sites
		Warwick-Edinburgh Mental Well-being Scale (WEMWBS) ^©^ [55]	All sites
Physical activity level and behavior		Modified Recent/Youth/Children´s Physical Activity Questionnaire (mRPAQ/mYPAQ/mCPAQ), *adapted from the Recent Physical Activity Questionnaire (RPAQ)* [56]	All sites
		Half-structured interview on physical activity and behavior	All sites
*Exercise capacity outcomes*			
Motor function		Quick Motor Function Test	All sites
Strength	Leg Strength	Handheld dynamometer	All sites
		Sit to stand test	All sites
		Leg extension machine	MBBM, UEM
	Arm strength	Handheld dynamometer	All sites
		Biceps curls (5 RM)	All sites
		Medicine ball shot	All sites
Endurance	Functional Capacity	Six Minute Walk Test (6MWT)	All Sites
	Cardiorespiratory Fitness	CPET using a cycling ergometer	UMC-Mainz, MBBM, UEM, INT, RegionH
	Cardiorespiratory Fitness	CPET using the YoYo-Test [57]	MBBM
		6-min cycling test (6MCT)	UMC-Mainz
	Muscle Oxygenation	Near infrared spectroscopy (NIRS)	MBBM
Flexibility		Sit and Reach Test	All Sites
		Goniometry	All Sites
Functional Mobility		Timed Up and Down Stairs Test (TUDS)	All Sites
*Body composition*			
Body composition		Waist-to-Hip Ratio	All Sites
		Bioelectrical impedance analysis (BIA)	All Sites
		Dual energy X-ray absorptiometry (DEXA)	UEM, INT, RegionH
	Skinfold Thickness	Plicometry	All Sites
*Safety outcomes*			
Serious exercise-related health complications (SERC)		Type and number of SERC	All sites

*Abbreviations*: *PedsQL™* Pediatric Quality of Life Inventory™, *mRPAQ* Modified Recent Physical Activity Questionnaire, *mYPAQ* Modified Youth Physical Activity Questionnaire, *mCPAQ* Modified Children’s Physical Activity Questionnaire, *MRScc* Mainzer Resilience Scale for Childhood Cancer, *BRS* Brief Resilience Scale, *CYRM-R* Child & Youth Resilience Measure-Revised, *GSE* General Self-Efficacy Scale, *PESE* Physical Exercise Self-Efficacy Scale, *WHO-5* World Health Organization (Five) Well-Being Index, *WEMWBS* Warwick-Edinburgh Mental Well-Being Scale, *RPAQ* Recent Physical Activity Questionnaire, *6MWT* Six Minute Walk Test, *CPET* Cardiopulmonary Exercise Testing, *NIRS* Near Infrared Spectroscopy, *TUDS* Timed Up and Down Stairs Test, *BIA* Bioelectrical Impedance Analysis, *DEXA* Dual Energy X-ray Absorptiometry

*Abbreviations* (Data Collection Sites): *INT* Fondazione IRCCS Istituto Nazionale dei Tumori, *MBBM* Fondazione Monza e Brianza per Il Bambino e La Sua Mamma, *RegionH* Region Hovedstaden, *UEM* Universidad Europea de Madrid, *UMC-Mainz* University Medical Center of the Johannes Gutenberg-University Mainz.

In addition to questionnaire- and exercise-based outcomes, blood samples will be collected from participants aged four years and older with solid tumors (excluding leukemia and lymphoma) at three recruiting centers (UMC-Mainz, UEM, INT) for biomarker analysis. The samples will be analyzed for immunological and epigenetic parameters by UEM and DSHS Koeln.

Outcome comparisons will primarily focus on the pre-test (T0) versus post-test (T1) assessments, with additional analyses conducted at subsequent follow-up time points.

A detailed overview on outcome measures and study time points is provided in Fig. 1 and in the SPIRIT Figure (see Additional file 2).

### Technology

The FORTEe trial integrates a range of technological tools to enhance the intervention, particularly for the exercise group. As part of the FORTEe project, the"FORTEe– Get strong"mobile app has been developed to provide educational and exercise- and health-related content along with a comprehensive exercise catalogue.

To further support exercise training, a novel AR application, the FORTEe AR app, has been developed. It enables participants to perform exercise sessions with an animated avatar. The FORTEe AR app is used both for home-based training and to enhance in-hospital exercise sessions at selected study sites (UMC-Mainz, UKHD, MBBM, and INT).

In addition, at specific study sites (UMC-Mainz, UKHD, MBBM, and INT), an interactive 2D screen (the Pixformance station) may be employed during in-hospital sessions. This system features an integrated camera that analyzes body segments in real time and provides immediate feedback on exercise execution and movement quality.

Furthermore, telehealth-supervised sessions are conducted to further support participants during their home-based training.

### Data management and monitoring

Within the FORTE trial, every precaution is taken to protect the privacy of research subjects and the confidentiality of their personal information and data. Principles for the protection of the participants’ personal data is described in more detail in the FORTEe Data Protection Concept. Data Protection obligations and responsibilities within the FORTEe Consortium are defined in a Joint Controllership Agreement (Article 26 GDPR). Furthermore, Data Processing Agreements (Article 28 GDPR) with data-processing parties have been concluded. A data protection impact assessment (art. 35 GDPR) was performed prior to the start of the clinical trial. In order to guarantee appropriate handling of the collected data, a detailed data management plan (DMP) was developed prior to the start of the clinical trial.

Clinical and study data, including records of any (serious) exercise-related health complications, will be collected via electronic case report forms and stored in the online MARVIN database. Data Traceability is ensured by an integrated audit trail. The database complies with all relevant laws and regulations, including Good Clinical Practice and the European General Data Protection Regulation (EU) 2016/679. MARVIN supports data import and export functionalities and employs state-of-the-art security measures to protect both study data and participants’ personal data. Data management in FORTEe will adhere to the FAIR principles, ensuring that all data are Findable, Accessible, Interoperable, and Reusable. To facilitate knowledge exchange with the scientific community, relevant data will be deposited in public repositories and made publicly available following an embargo period.

To ensure data security in FORTEe, a Data and Safety Monitoring Committee (DSMC) has been established. It comprises the trial statistician and three experts in data and study management. The DSMC reviews serious exercise related events, assesses potential statistical issues—such as selection bias, adherence concerns, or failure to follow up—and advises the Steering Committee (SC) and trial leadership on critical safety matters.

To ensure the proper execution of exercise testing, the exercise intervention, and documentation in accordance with the study protocol, monitoring visits are conducted at defined intervals by designated study personnel.

### Sample size

The study is powered for comparisons within each of three strata: leukemia/lymphoma, CNS tumors, and other tumors. Based on the pilot MUCKI trial [36] at the coordinating center, the expected distribution is 45% of patients in the leukemia/lymphoma stratum, 25% in the CNS tumor stratum, and 30% in the other tumors stratum. At the time of sample size calculation, the study was designed with these three strata based on cancer diagnosis. For the purposes of randomization, an additional stratum specifically for malignant bone tumors was later introduced, increasing the total number of strata for randomization to four. This change was implemented during the preparation of the final study protocol in consent with the DSMC. Importantly, this modification does not affect the original sample size calculation or the statistical power, which remains based on the three strata originally considered.

Each stratum’s comparison between the active intervention and the corresponding control group is weighted equally, with a two-sided significance level of 1.67% applied for each comparison. With 360 evaluable patients, the anticipated statistical power is 92% for the leukemia/lymphoma stratum (2 × 81 = 162 patients), 66% for the CNS tumor stratum (2 × 45 = 90 patients), and 82% for the other tumors stratum (2 × 63 = 126 patients). Accounting for an estimated 20% dropout rate, a total of 450 patients will be randomized.

### Statistical analysis

Statistical methods are defined within the trial’s statistical analysis plan. The primary endpoint is the change in CRF from baseline to T1, assessed using the PedsQL™ 3.0 Multidimensional Fatigue Scale. The primary analysis will compare the exercise and control groups using an analysis of covariance (ANCOVA) model, with the intervention group, tumor entity, and center treated as fixed factors, and baseline CRF score included as a covariate. Additionally, pairwise comparisons between the exercise and control groups within each tumor stratum will be performed using two-sample t-tests on the change from baseline, derived from the ANCOVA model.

The primary analysis population will follow the intention-to-treat principle, meaning all randomized participants will be analyzed in their originally assigned groups, regardless of adherence, withdrawal, or protocol deviations occurring after randomization. A per-protocol population will also be defined, including all randomized participants who do not have major protocol violations. The primary hypothesis test will be conducted at a two-sided overall significance level of 5%.

A sensitivity analysis will be conducted on the per-protocol population to assess the robustness of the intervention effects on CRF, HRQoL, and exercise capacity. Missing data will be handled using multiple imputation methods, where appropriate. All variables will be summarized using descriptive statistics, and exploratory p-values will be reported.

Subgroup analyses by tumor entity, treatment intensity, cancer treatment and age group are planned. No interim analysis is planned for this trial.

### Roles and responsibilities

The FORTEe project is coordinated by UMC-Mainz, which serves as the legal entity and intermediary between the consortium and the European Commission. The coordinating center is responsible for overseeing project implementation, chairing both the General Assembly (GA) and the Steering Committee, and acting as the primary communication link between all involved parties.

The Clinical Trial Management Committee– comprising MBBM, UMC-Mainz and UEM- plays a key role in coordinating and supervising the clinical trials.

The SC, consisting of all work package leaders, is responsible for the successful execution of the project in terms of schedule, budget, and scientific quality.

The GA serves as the highest decision-making body within the FORTEe consortium. It includes one voting representative from each project partner, as well as non-voting researchers involved in the project. The GA is responsible for making strategic decisions, particularly regarding changes to project scope, finances, and intellectual property rights.

The Scientific and Ethical Advisory Board (SEAB), comprising external experts, provides independent guidance to ensure scientific rigor and ethical integrity. SEAB supports project quality through annual GA meetings, progress reviews, and identification of emerging ethical issues.

### Dissemination

A comprehensive dissemination and exploitation strategy including publication rules have been developed to ensure that FORTEe’s findings are accessible and effectively communicated to key stakeholders, including the scientific, clinical, and public health communities, guideline committees, and the general public.

## Discussion

Exercise oncology is not yet a standard component of care in pediatric oncology, despite evidence showing its positive effects on both psychosocial aspects and physical performance [24–32, 36, 58]. Children and adolescents who have the opportunity to participate in oncology exercise programs report that exercise therapy provides them with a means to actively contribute to their treatment. This active involvement can foster increased self-efficacy and self-esteem [59, 60]. To fully assess and evaluate the benefits of exercise oncology, randomized controlled trials RCTs are essential. However, only a limited number of RCTs have been conducted on exercise interventions in pediatric oncology during active cancer treatment [25, 26, 32, 61–68]. Nevertheless, generalizable conclusions remain challenging due to small sample sizes and diverse study designs.

To advance and unify the emerging field of pediatric exercise oncology across European countries, the FORTEe project and trial bring together leading experts from seven European nations. This collaborative effort aims to expand the expertise in the field and develop specific exercise training and testing protocols, which will be implemented with the aid of digital technologies.

### Strengths of the study design

The FORTEe trial will be one of the largest interventional studies in pediatric exercise oncology, with a planned sample size of 450 patients. This large sample size will increase the statistical power of the trial, allowing for more robust analyses of the effectiveness of the intervention in a diverse population of children, adolescents, and young adults undergoing cancer treatment. By enrolling participants from multiple centers across Europe, the trial aims to reflect a wide range of clinical practices and patient characteristics, which will contribute to the generalizability of the results. This diversity will help ensure that the findings are relevant across different healthcare systems and types of cancer treatment.

The FORTEe trial is a multicenter, randomized controlled design that incorporates both personalized and standardized elements of exercise therapy. One of the key strengths is the personalization of the exercise intervention, which adapts the dosage (type, intensity, duration) based on individual patient needs and treatment phases. This tailored approach aims to address the heterogeneity in patient responses to both cancer treatment and exercise. The inclusion of remote exercise supervision through digital platforms and telehealth solutions enhances the feasibility of the intervention, providing accessibility to patients across various clinical settings (e.g., inpatient, outpatient, and home settings).

### Limitations and challenges

Despite its strengths, the trial does face several limitations. One of the primary challenges is blinding; due to the nature of the intervention (exercise), blinding of participants and personnel is not feasible, which could introduce bias in the reporting of subjective outcomes such as CRF and HRQoL. Additionally, while the use of remote tools enhances accessibility, it may also introduce variability in adherence and the quality of exercise execution, which could influence outcomes.

Another potential limitation is the diversity of treatment protocols across participating centers. Variations in cancer treatment regimens, such as chemotherapy intensity and duration, could affect patient response to the exercise intervention. However, this challenge is being addressed by standardizing exercise protocols and using a comprehensive functional assessment system to tailor exercise to individual patient needs.

### Implications and future directions

Once completed, the FORTEe trial has the potential to provide robust evidence on the effects of exercise therapy. Subsequently, the aim is to implement pediatric exercise oncology as an evidence-based treatment option for all pediatric cancer patients, and ultimately to integrate it as a standard in clinical practice worldwide.

## Supplementary Information

Additional file 1. SPIRIT 2013 Checklist for the FORTEe Trial Protocol.

Additional file 2. Study design and timeline for the FORTE trial in accordance with SPIRIT 2013 guidelines.

Additional file 3. Recommendations for medical clearance/ reasons for adapting exercise.

Additional file 4. (Serious) Exercise-Related health Complications (SERCs).

Additional file 5. Inclusion and exclusion criteria of the FORTEe trial (Clinial Study Protocol, V1.4– 2023/05/01).

Additional file 6. Supportive care services across trial sites.

## Data Availability

No datasets were generated or analysed during the current study.

## Abbreviations

AR: Augmented reality
CLB: Centre de Lutte Contre le Cancer Leon Berard
CNS: Central nervous system
CONSORT: Consolidated Standards of Reporting Trials
CPET: Cardiopulmonary exercise testing
CRF: Cancer-related fatigue
GA: General Assembly
GDPR: General Data Protection Regulation
HRQoL: Health-related quality of life
INT: Fondazione IRCCS Istituto Nazionale dei Tumori
MBBM: Fondazione Monza e Brianza per Il Bambino e La Sua Mamma
PedsQL™: Pediatric Quality of Life Inventory™ Multidimensional Fatigue Scale
RCT: Randomized, controlled trial
SEAB: Scientific and Ethical Advisory Board
SERC: Serious Exercise-Related health Complication
SPIRIT: Standard Protocol Items: Recommendations for Interventional Trials
