# Supplementary material for: Get strong to fight childhood cancer - an exercise intervention for children and adolescents undergoing anti-cancer treatment (FORTEe): Rationale and design of a randomized controlled exercise trial
Source: BMC Cancer. 2025 Aug 7;25:1275. doi: 10.1186/s12885-025-14489-y (PMC12330123; doi:10.1186/s12885-025-14489-y)
Supplement: Supplementary file 2 — Additional file 2. Study design and timeline for the FORTE trial in accordance with SPIRIT 2013 guidelines. [file 12885_2025_14489_MOESM2_ESM.pdf]

Study design and timeline for the FORTE trial in accordance with SPIRIT 2013 guidelines (39). The table outlines key trial activities and time points, from enrollment and eligibility screening through baseline assessments, interventions, and follow-up evaluations. Each row represents specific procedures (e.g., informed consent/enrolment, allocation/randomization, exercise sessions, questionnaires, and biological sampling) mapped to their respective time points (T–1, T0, T1, etc.). This layout ensures clarity on when and how each procedure is conducted throughout the trial.

**During the intervention phase, CRF and resilience will be assessed additionally every two weeks via questionnaires. An assessment of CRF and resilience via questionnaires will be performed twice (every four weeks) from T1 until T2.*

		**STUDY PERIOD**						
	**Enrolment**	**Before start of anti-cancer treatment**	**Baseline**	**Allocation**	**Post-allocation**			
**TIMEPOINT**	***-t₁***	***T-1***	***T0***	***0***	***T1***	***T2***	***T3***	***T4***
**ENROLMENT:**								
**Eligibility screen**	X							
**Informed consent**	X							
**Allocation**				X				
**INTERVENTIONS:**								
Exercise Intervention				↔				
**ASSESSMENTS:**								
Questionnaire programme *			X	↔		X	X	X
Exercise testing			X		X	X		
Blood sampling		X	X		X			

References

39. Chan AW, Tetzlaff JM, Altman DG, Laupacis A, Gotzsche PC, Krleza-Jeric K, et al. SPIRIT 2013 statement: defining standard protocol items for clinical trials. Ann Intern Med. 2013;158(3):200-7.
