# Supplementary material for: Get strong to fight childhood cancer - an exercise intervention for children and adolescents undergoing anti-cancer treatment (FORTEe): Rationale and design of a randomized controlled exercise trial
Source: BMC Cancer. 2025 Aug 7;25:1275. doi: 10.1186/s12885-025-14489-y (PMC12330123; doi:10.1186/s12885-025-14489-y)
Supplement: Supplementary file 3 — Additional file 3. Recommendations for medical clearance/ reasons for adapting exercise. [file 12885_2025_14489_MOESM3_ESM.pdf]

Recommendations for Medical Clearance and Reasons for Adapting Exercise

This document summarizes expert consensus and systematic literature review findings to guide exercise professionals and medical staff in the FORTEe trial. It highlights key conditions—such as bleeding risk, anemia, infections, pain, tumor characteristics, cardiovascular and respiratory issues, post-procedural status, and other co-morbidities—that necessitate individualized adjustments or contraindicate exercise sessions.

General information

- Before the start of an exercise session, the medical clearance for exercise training must be obtained (e.g. by the treating physician/oncologist/sports medicine physician).
- This guideline is based on both a systematic literature review and the expert consensus of the FORTEe Consortium. It is intended to serve as a guide for the exercise and medical professionals of the FORTEe staff during the exercise intervention of the FORTEe clinical trial.

The physician and the exercise professional shall decide jointly and individually for each patient and prior to each session which type of exercise training is possible in the individual case.

Bleeding risk & Platelet count

- Children with platelet counts below 10,000 per µL should not exercise.
- In case of platelet levels below 30,000/µl, the exercise session should be adjusted individually.
- Nevertheless, the individual bleeding risk must be considered, especially with regard to:
  - Risk of intracerebral haemorrhage
  - Signs of haemorrhage
  - Co-existing infection
  - Co-existing hyperleukocytosis
  - Co-existing coagulation abnormalities
  - Platelet count dynamics (e.g. rapid fall of platelet count)

Anemia

- If Haemoglobin < 8g/dl, the exercise session should be adjusted individually.
- In patients with symptomatic anaemia (e.g. dizziness, dyspnoea), the exercise session should be adjusted individually.

Fever and infections

- No exercise with fever > 38,0°C (even if normalised/treated by fever-reducing medication).
- No exercise in case of a severe infection or systemic infection.
- In case of a mild infection (without fever) or in case of local infections, the exercise session should be adjusted individually.

General disorders and pain

- No exercise in case of severe pain.
- In case of mild pain or dizziness, the exercise session should be adjusted individually.

Bone tumours & risk of fractures

- In patients with bone tumours or metastases, the affected region should not be loaded and treated with special care (in consultation with the treating physician). PARTICULAR CAUTION is needed in patients with tumours and metastases of the spine.

Central nervous system tumours

- Patients with central nervous system tumours (brain and spinal tumours) are at higher risk of falling and injury.

Thus, the exercise session should be adjusted individually with regard to:

  - Paresis/Ataxia
  - Cognitive impairment
  - Risk of seizure

Cardiovascular disorders

- Patients with cardiovascular disorders are at higher risk for lethal complications, especially patients with

the following conditions should not exercise:

- Acute heart failure (especially when symptomatic or decompensated)
- Acute or active myocarditis or pericarditis or endocarditis
- Cardiac arrhythmia (especially when causing symptoms or hemodynamic compromise)
- Severe arterial or pulmonary hypertension
- Large vessel thrombosis/deep vein thrombosis/embolisms
- Circulatory instability with need of administration of e.g. catecholamines

Respiratory insufficiency

- No exercise in case of a respiratory insufficiency.

Post-operative & post-interventional situation

- Particular caution is needed in the following situations:
  - after biopsy/lumbar puncture
  - after surgical interventions
  - existing wound-drainages
- If the healing of surgical wounds has not yet been completed, the exercise session should be individually adjusted (no local loading).

Chemotherapy

- If possible, exercise sessions scheduled during ongoing/running intravenous chemotherapy should be avoided, e.g. due to the risk of dislocation of the central venous catheter.

Particular caution is needed in the following situations:

- application of cardiotoxic chemotherapy (e.g. anthracyclines)

Radiotherapy

No exercise during total body irradiation and mediastinal or cardiac-directed irradiation (up to 72 hours after the end of irradiation).

Nausea/vomiting

- In case of nausea/vomiting, the exercise session should be adjusted individually.

Co-Morbidities/ pre-existing conditions

Particular caution is needed in the following situations:

- Acute or chronic disorder that may be aggravated by exercising, e.g.:
  - renal failure
  - metabolic disease (especially when uncontrolled), e.g. diabetes
- Physical disability or mental impairment that would preclude safe and adequate test performance or exercising
