# Supplementary material for: Get strong to fight childhood cancer - an exercise intervention for children and adolescents undergoing anti-cancer treatment (FORTEe): Rationale and design of a randomized controlled exercise trial
Source: BMC Cancer. 2025 Aug 7;25:1275. doi: 10.1186/s12885-025-14489-y (PMC12330123; doi:10.1186/s12885-025-14489-y)
Supplement: Supplementary file 4 — Additional file 4. (Serious) Exercise-Related health Complications (SERCs). [file 12885_2025_14489_MOESM4_ESM.pdf]

(Serious) Exercise-Related health Complications (SERCs)

The reporting of (Serious) Exercise-Related health Complications (SERCs) in the FORTEe trial is structured based on the Common Terminology Criteria for Adverse Events (CTCAE) (65), utilizing its established code groups and specific codes to ensure standardized and consistent reporting of adverse events.

<u>FORTEe SERC Code Groups</u>

SERC Code group	• Cardiac disorder (CD)<br>• General disorders and administration side conditions (GDSC)<br>• Injuries and procedural complications (IPC)<br>• Musculoskeletal and connective tissue disorder (MTD)<br>• Nervous system disorder (NSD)<br>• Respiratory, thoracic and mediastinal disorder (RTMD)<br>• Vascular disorder (VD)<br>• Gastrointestinal disorders

<u>FORTEe SERC Codes</u>

SERC Codes - Cardiac disorder (CD)	• Chest pain – cardiac<br>• Palpitations<br>• Other cardiac disorder
SERC Codes - General disorders and administration side conditions (GDSC)	• Pain<br>• Non-cardiac chest pain<br>• Malaise<br>• Other general disorders
SERC Codes - Injuries and procedural complications (IPC)	• Fracture<br>• Bruising<br>• Fall<br>• Other injuries
SERC Codes - Musculoskeletal and connective tissue disorder (MTD)	• Arthralgia<br>• Back pain<br>• Bone pain<br>• Myalgia (muscular pain)<br>• Generalized muscle weakness<br>• Muscle cramp<br>• Other musculoskeletal disorders
SERC Codes - Nervous system disorder (NSD)	• Dizziness<br>• Headache<br>• Muscle weakness<br>• Presyncope<br>• Syncope<br>• Seizure<br>• Other nervous system disorder
SERC Codes - Respiratory, thoracic and mediastinal disorder (RTMD)	• Hypoxia<br>• Dyspnea<br>• Other RTMD

SERC Codes - Vascular disorder (VD)	• Hematoma Bleeding<br>• Dislocation central (venous) catheter<br>• Other CD disorder
SERC Codes - Gastrointestinal disorders	• Abdominal pain<br>• Nausea / Vomiting

<u>FORTEe SERC Grades</u>

- Grade 3 (Severe): Severe or medically significant, but not immediately life-threatening; hospitalisation or prolongation of hospitalisation indicated; disabling
- Grade 4 (Life-threatening): Life-threatening consequences; urgent intervention indicated
- Grade 5 (Death): Death related to SERC

References

65. U.S. Department of Health and Human Services NIoH, National Cancer Institute. Common Terminology Criteria for Adverse Events (CTCAE) v5.0 [Web]. Bethesda, MD: National Institutes of Health; 2017 [updated November 27, 2017. 5.0]. Available from: https://ctep.cancer.gov/protocoldevelopment/electronic_applications/ctc.htm.
