# Supplementary material for: Get strong to fight childhood cancer - an exercise intervention for children and adolescents undergoing anti-cancer treatment (FORTEe): Rationale and design of a randomized controlled exercise trial
Source: BMC Cancer. 2025 Aug 7;25:1275. doi: 10.1186/s12885-025-14489-y (PMC12330123; doi:10.1186/s12885-025-14489-y)
Supplement: Supplementary file 5 — Additional file 5. Inclusion and exclusion criteria of the FORTEe trial (Clinial Study Protocol, V1.4– 2023/05/01). [file 12885_2025_14489_MOESM5_ESM.pdf]

Inclusion and exclusion criteria of the FORTEe trial (Clinial Study Protocol, V1.4 – 2023/05/01)

Inclusion criteria	Exclusion criteria
First/initial diagnosis of a **primary oncologic disease** according to the International Classification of Childhood Cancer (ICCC), requiring chemotherapy and/or radiation therapy **or**<br>First/initial diagnosis of a **relapsed oncologic disease** according to the International Classification of Childhood Cancer (ICCC), requiring chemotherapy and/or radiation therapy **or**<br>First/initial diagnosis of a **secondary oncologic disease** according to the International Classification of Childhood Cancer (ICCC), requiring chemotherapy and/or radiation therapy	Oncologic disease (primary/relapsed/secondary) according to the International Classification of Childhood Cancer (ICCC), requiring neither chemotherapy nor radiation therapy or solely requiring surgery
Age: **≥4 to ≤21 years** (completed 4 years of age to not completed 22 years of age)	
Planned or started anti-cancer treatment (**chemo- and/or radiotherapy**) at one of the recruiting centers of the FORTEe trial	Reasons that already exist or are foreseeable at the time of study enrolment, due to which the patient will not have access to the exercise intervention and/or to the pre-test and/or to the post-test (e.g. foreseeable or planned, permanent change of treatment center).
The patient is assessed by the treating team (pediatric oncologist, exercise professional etc.) as suitable to participate in the trial, e.g. due to medical or psychological reasons.	The patient is assessed by the treating team (pediatric oncologist, exercise professional etc.) as unsuitable to participate in the trial, e.g. due to medical or psychological reasons
Existing **informed consent (or assent)** to participate in the study<br>• For minor patients (≤18 years): The legal guardians assume that the child/adolescent is able to freely decide on participation or to consent to participation on the basis of the information received and have given written consent for participation in the study after detailed information and a time to consider (i.e. at least 24 hours). In addition to the informed consent given by the legal guardians, in adolescents ≥16 years of age, written consent to participate in the study is mandatory after detailed age appropriate information and after a time to consider. Documented assent is sought from all minor patients after age appropriate information. In the case of children > 6 years of age, this should, if possible, also be given as written informed consent.<br>• For adult patients (≥18 years): Existing written consent to participate in the study after detailed information and after a time to consider (i.e. at least 24 hours).	After detailed information and, if necessary, having time to consider, the patient (≥ 16 years of age) did not agree to give written informed consent to participate in the trial.<br>In the case of minor patients (<16 years of age): The legal guardians do not assume that the child/adolescent is able to freely decide on participation or to consent to participation on the basis of the information received, and do not consent to this themselves.<br>In case of minors capable of giving consent*: Lacking documented assent/ written informed consent.<br><br>** in general minors are considered as capable of giving consent at the age of 14*
The patient (and the legal guardian) has/have sufficient knowledge of the respective national or English language so that it is possible to carry out the informed consent as well as interviews (in age-appropriate language).	The patient (and the legal guardians) has/have insufficient knowledge of the respective national or English language, so that it is not possible to carry out both the informed consent and interviews (in age-appropriate language).

The patient is not in a terminal phase of the disease.<br>*("Terminal" here refers to the last phase of the disease before dying, but does not automatically refer to patients who are defined and cared for as "palliative". Despite their assumed increased vulnerability, a general exclusion of palliative patients from the trial would provoke an inadequate bias of the study population and at the same time deny these patients a potentially enriching opportunity to participate.)*	The patient is in a terminal phase of the disease.<br>*("Terminal" here refers to the last phase of the disease before dying, but does not automatically refer to patients who are defined and cared for as "palliative". Despite their assumed increased vulnerability, a general exclusion of palliative patients from the trial would provoke an inadequate bias of the study population and at the same time deny these patients a potentially enriching opportunity to participate.)*
