# Supplementary material for: Get strong to fight childhood cancer - an exercise intervention for children and adolescents undergoing anti-cancer treatment (FORTEe): Rationale and design of a randomized controlled exercise trial
Source: BMC Cancer. 2025 Aug 7;25:1275. doi: 10.1186/s12885-025-14489-y (PMC12330123; doi:10.1186/s12885-025-14489-y)
Supplement: Supplementary file 6 — Additional file 6. Supportive care services across trial sites. [file 12885_2025_14489_MOESM6_ESM.pdf]

**Supportive care services provided as part of usual care for both the FORTEe control and intervention groups at each trial site.**

The table shows which services were available at each site, with "x" indicating the availability of the service.

| | **Supportive care services offered as part of usual care at each FORTEe trial site** | | | | | | | | | |
|---|---|---|---|---|---|---|---|---|---|---|
| **Type of Supportive Care** | UMC-Mainz | UKHD | UKESSEN | MBBM | CLB | OBU | RegionH | UEM | INT | UKCL |
| Psychology services | x | x | x | x | x | x | x | x | x | x |
| Social services | x | x | x | x | x | x | x | x | x | x |
| Education/ kindergarden/ activity or play therapy | x | x | x | x | x | x | x | x | x | x |
| Music therapy | x | x | x | x | x | | | x | | x |
| Art therapy | x | | x | x | x | | | x | x | |
| Physiotherapy | x | x | x | x | x | x | x | x | x | x |
| Relaxation techniques (e.g. breathing techniques, autogenic training, progressive muscle relaxation, imagination) | x | x | | | x | | | | | x |
| Occupational therapy/ergotherapy | x | | x | | | x | x | | x | x |
| Dietary advice/nutrition consultant | x | | x | x | x | x | x | x | x | x |

**Abbreviations:** UMC-Mainz: University Medical Center of the Johannes Gutenberg-University Mainz; UKHD: Heidelberg University Hospital; UKESSEN: University Hospital Essen; MBBM: Fondazione Monza e Brianza per Il Bambino e La Sua Mamma; CLB: Centre de Lutte Contre le Cancer Leon Berard; OBU: Oxford Brookes University; RegionH: Region Hovedstaden; UEM: Universidad Europea de Madrid; INT: Fondazione IRCCS Istituto Nazionale dei Tumori; UKCL: University Medical Center Ljubljana.
